# Virginia Medical Monthly

**Published:** 1879-02

**Authors:** 


*Virginia Medical Monthly.*—

Attention has been called in a late number to this sterling magazine. The article in the October No., on the Etiology of Typhoid Fever, by Dr. Bramlette, of Va., and although iconoclastic, is replete with information, and will form valuable material for generalizing by some one. Would it not be as well if more work of this sort were done, more facts gathered, and fewer dogmatic conclusions made without the facts?

The paper by Dr. J. J. Caldwell, of Baltimore, in the same No. is a valuable review of the neuroses of the pneumogastric and sympathetic nerves, and of the vaso and sweat centres; with appropriate therapy. This paper has attracted the attention of many distinguished pathologists, and its author in it shows an intimate acquaintance not only with the literature of this subject but also with the treatment of lesions of these nerves.

Dr. Wm. Selden, of Norfolk, Va., contributes an article on opium poisoning relieved by immersion of the feet of the narcotized person in hot water. Would this treatment, we suggest, be of any service in anæsthetic accident?

This Journal, (*Va. Medical,*) is contributed to by writers from Massachusetts to Wisconsin, from California to Mississippi, from New York to Texas; sixty-seven contributors, representing twenty-one States and territories, make up the list of the first seven months of the fifth volume, and the journal is only that old! Edited by Dr. Landon B. Edwards, Richmond, Va.

